# Mental health and cerebellar volume during adolescence in very-low-birth-weight infants: a longitudinal study

**DOI:** 10.1186/s13034-016-0093-8

**Published:** 2016-03-16

**Authors:** Violeta L. Botellero, Jon Skranes, Knut Jørgen Bjuland, Gro C. Løhaugen, Asta Kristine Håberg, Stian Lydersen, Ann-Mari Brubakk, Marit S. Indredavik, Marit Martinussen

**Affiliations:** Department of Laboratory Medicine, Children’s and Women’s Health, Faculty of Medicine, Medical Technology Research Center, Norwegian University of Science and Technology, P.O. Box 8905, 7491 Trondheim, Norway; Department of Neuroscience, Norwegian University of Science and Technology, Trondheim, Norway; Regional Center for Child and Youth Mental Health and Child Welfare, Norwegian University of Science and Technology, Trondheim, Norway; Department of Pediatrics, Sørlandet Hospital, Arendal, Norway; Department of Medical Imaging, St. Olav’s University Hospital, Trondheim, Norway; Department of Pediatrics, St. Olav’s University Hospital, Trondheim, Norway; Department of Child and Adolescent Psychiatry, St. Olav’s University Hospital, Trondheim, Norway; Department of Gynecology and Obstetrics, St. Olav’s University Hospital, Trondheim, Norway

**Keywords:** Cerebellum, Preterm, Psychiatric disorders, MRI, Very low birth weight, Mental health

## Abstract

**Background:**

Preterm birth at very low birth weight (VLBW) poses a risk for cerebellar abnormalities and increased psychiatric morbidity compared with reference populations. We aimed to study cerebellar volumes (grey and white matter; GM, WM) and mental health in VLBW individuals and controls at 15 and 19 years of age, as well as changes between the two time points.

**Methods:**

Forty VLBW (≤1500 g) and 56 control adolescents were included in the study at 15 years of age, and 44 VLBW and 60 control adolescents at 19 years of age. We had longitudinal data for 30 VLBW participants and for 37 controls. Clinical diagnoses were assessed following the schedule for affective disorders and schizophrenia for school-age children (KSADS). Psychiatric symptoms and function were further investigated with the Achenbach System of Empirically Based Assessment (ASEBA), ADHD Rating Scale-IV and the children’s global assessment scale (CGAS). An automatic segmentation of cerebellar GM and WM volumes was performed in FreeSurfer. The MRI scans were obtained on the same 1.5T scanner at both ages.

**Results:**

The VLBW group had higher rates of psychiatric disorders at both ages. Cerebellar growth trajectories did not differ between VLBW adolescents and controls, regardless of psychiatric status. However, VLBW adolescents who had a psychiatric diagnosis at both ages or developed a psychiatric disorder from 15 to 19 years had maintained smaller cerebellar WM and GM volumes than controls and also smaller volumes than VLWB adolescents who were or became healthy in this period. Moreover, there were no differences in cerebellar WM and GM volumes between controls and those VLBW who were healthy or became healthy. In the VLBW group, cerebellar WM and GM volumes correlated positively with psycho-social function at both 15 and 19 years of age, and smaller GM volumes were associated with inattention at 15 years.

**Conclusions:**

Smaller cerebellar volume in adolescents born very preterm and with VLBW may be a biomarker of increased risk of psychiatric problems in young adulthood.

**Electronic supplementary material:**

The online version of this article (doi:10.1186/s13034-016-0093-8) contains supplementary material, which is available to authorized users.

## Background

Over the years, very preterm born children (<32 weeks gestation) have better survival rates [1] and improved outcome [2]. It is, however, of concern that an increased risk of psychiatric problems has been reported in preterm-born individuals (<37 weeks gestation), especially anxiety symptoms and disorders, attention-deficit/hyperactivity disorder (ADHD) and autism spectrum traits and disorders (ASD) [3–5].

The cerebellum is of particular interest in the preterm born due to its extensive development during the third trimester of gestation. Indeed, during this period it surpasses the growth rate of the cerebral hemispheres [6]. For the very preterm born infant, the extensive development of the cerebellum takes place in an extra uterine environment, where respiratory problems, infections and nutritional challenges may influence cerebellar development. Cerebellar injuries (hemorrhage, infarction) and mal/underdevelopment following premature birth occur more frequently than previously thought [7, 8]. It has been proposed that cerebellar involvement may play a central role in cognitive, mental health, and socialization deficits found later in life in this population [9, 10]. In the general population, the cerebellum has been associated with psychiatric problems such us mood disorders, anxiety problems, schizophrenia, ASD and attention problems [11]. The underlying pathophysiology still remains unknown. However, it has been proposed that the cerebellum might serve as modulatory [12–14] and timing station [15–17] for integrating [18] brain processes, due to its extensive connections with the whole brain [14, 19–22]. Projections from the cerebellum to the cerebral cortex constitute the cerebello-thalamo-cortical (CTC) pathway [23, 24], and early disruption of the cerebellar circuitry development has been positively correlated with ASD, attention deficit and emotional problems [25]. Injury to the immature cerebellum could affect neurologic function through mechanisms that interfere with later development of remote regions of the cerebral cortex [26].

Some studies have shown cerebellar abnormalities to be associated with psychiatric symptoms in preterm children. In a retrospective, case–control study, preterm children who had perinatal cerebellar hemorrhage presented a higher prevalence of deficits in cognition, communication, and social and behavioral function at 2–3 years of age than preterm peers without cerebellar pathology [10]. In another MRI study with very preterm participants, total cerebellar volume reduction from adolescence to adulthood was associated with having more psychiatric symptoms [27]. However, these studies used questionnaires to assess mental health problems and did not differentiate between cerebellar gray (GM) and white matter (WM).

Our research group has studied preterm born adolescents with very low birth weight (VLBW ≤ 1500 g) and controls during adolescence. At 15 years of age, the VLBW children had smaller cerebellar WM volumes compared with controls [28], and they had increased rates of psychiatric symptoms and diagnoses assessed with questionnaires and clinical interview [3, 4]. At 19 years of age, the VLBW group still had smaller cerebellar WM volumes than term-born peers [29] and more psychiatric problems [5]. During adolescence they also displayed a trend towards increasing psychiatric morbidity [5]. However, cerebellar growth rate did not differ from controls [29].

Based on these findings, we aimed to study the relationship between cerebellar volumes and psychiatric symptoms and diagnoses at 15 and 19 years of age. Our main hypothesis was that reduced cerebellar volumes would be associated with higher rates of psychiatric symptoms and diagnoses at both 15 and 19 years. Furthermore, we hypothesized that small cerebellar volume was associated with increased risk of developing psychiatric problems during adolescence.

In this article, we report an association between persistent smaller cerebellar volumes and psychiatric symptoms during adolescence in children born preterm and with VLBW.

## Method

### Participants

**Fig. 1:**
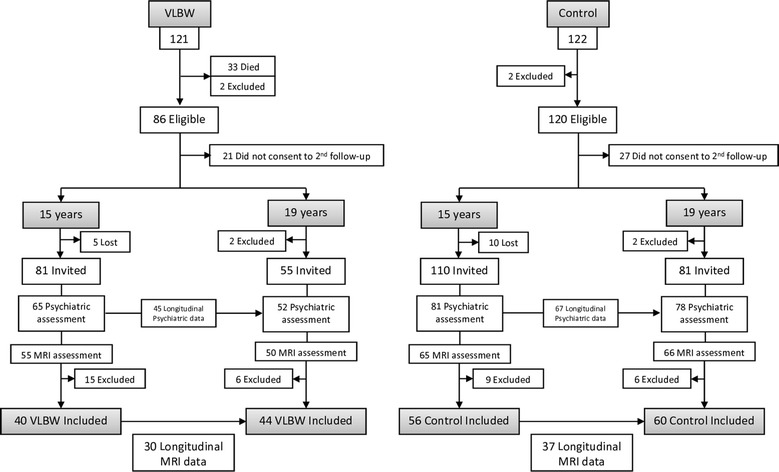
Chart that illustrates the composition of the VLBW and control groups at the two measurement points

We studied, from 15 to 19 years of age, a hospital based cohort of VLBW infants who were admitted to the neonatal intensive care unit at the Trondheim University Hospital (Norway) in 1986–1988 and an age-matched group of controls recruited among term-born children from the same geographical area with birth weight ≥10th percentile for gestational age [28] (Fig. 1). For the present study, 81 VLBW adolescents and 110 controls were invited at the age of 15. Of them, 55 VLBW and 65 control participants underwent MRI examination and psychiatric assessment. At the age of 19, 55 VLBW adolescents and 81 controls were invited. Of them, 50 VLBW and 66 control participants underwent MRI examination and psychiatric assessment. We included subjects who had valid MRI evaluations at least at one of the measuring points. Images of some participants were discarded from the MRI assessment due to dental brace artifacts and poor MRI quality due to movement. In total, 40 VLBW adolescents and 56 controls were included at 15 years, and 44 VLBW adolescents and 60 controls at the age of 19. There were, at both 15 and 19 years of age, a higher number of participants with psychiatric assessment than MRI scans. Thus, some of the participants had longitudinal psychiatric data, even though they did not have longitudinal MRI data. This enabled us to study diagnostic change in those participants with just one MRI scan (See Fig. 1 for details).

There were no significant differences between participants and non-participants with regard to maternal age at time of birth, birth weight, and gestational age.

Further details of the study population and design are given in previous publications [3–5, 28, 29].

### Ethics, consent and permissions

The Regional Committee for Medical Research Ethics approved the study protocol (project number: 78-00, May 2000 and 4.2005.2605) and the Data Inspectorate assigned the license for keeping a data register with personal information. Written informed consent was obtained from both adolescents and parents at the 15 years assessment, and from the participants at 19 years.

### MRI assessment

MRI was performed on the same 1.5 Tesla Siemens Symphony Sonata (Siemens AG, Erlangen, Germany) at St Olav’s University Hospital (Trondheim, Norway) at 15 and 19 years of age with Quantum gradients (30 mT/m) and a quadrature head coil. A structural T1-weighted magnetization prepared rapid acquisition gradient echo (MPRAGE) sequence was acquired with the following specifications: TR = 7.1 ms, TE = 3.45 ms, TI = 1000 ms, flip angle 7^o^, FOV 256 × 256, slab thickness 170 mm, slice thickness 1.33 mm, acquisition matrix 256 × 192 × 128, reconstructed to 256 × 256 × 128, giving a reconstructed voxel resolution of 1 × 1 × 1.33 mm, and acquisition duration of 8.5 min. Two MPRAGE sequences acquired at each time point (15 and 19 years) were registered to correct for head motion and averaged into a single image. FreeSurfer software package 5.3.0 (http://surfer.nmr.mgh.harvard.edu/) was used for the volumetric segmentation. This is an automated method of labeling human structures to extract GM and WM volumes for each participant’s entire brain [30, 31], and parcellating of the cortex of each participant [32, 33]. All processed images were visually inspected for accuracy of segmentation. Structures with obvious segmentation errors were rejected and no manual editing was performed to avoid introducing bias and increasing variances into the data set of MRI images. All images were processed with the longitudinal stream in FreeSurfer to enable longitudinal analyses [34–36] and to account for unbalanced time points [37]. For each participant, mean cerebellar volumes of GM and WM and estimated intracranial volume (eICV) were extracted and used in further analyses. The eICV volume is an indirect measure of the whole volume inside the human cranium except cerebellum, brain stem and ventricles.

### Clinical assessment

Parents and children were interviewed separately by senior clinicians at both follow-ups using the schedule for affective disorders and schizophrenia for school-age children (KSADS) [38]. Diagnoses were set according to the diagnostic and statistical manual of mental disorders, fourth edition (DSM-IV) [39] and categorized in three levels according to the KSADS scoring: (I) diagnoses (II) subclinical diagnoses (≥75 % of diagnostic criteria met, but not meeting criteria for full diagnosis), and (III) healthy. In order to study mental health over time, we divided the VLBW adolescents into two groups: persisting/developing diagnosis and healthy/becoming healthy. In the first group, we included those VLBW adolescents who had a psychiatric/subclinical diagnosis at both ages or developed one from 15 to 19 years. In the second group, we included VLBW adolescents who were healthy at both ages or became healthy from 15 to 19 years.

At the interview, general psycho-social functioning was rated according to the children’s global assessment scale (CGAS; scored from 1 to 100) [40]. To further assess psychiatric symptoms, the participants completed the Achenbach system of empirically based assessment (ASEBA); The youth self-report (YSR) at 15 years, and the adult self-report (ASR) at 19 years [41]. This is a screening instrument generating three composite scales: Total problems, internalizing and externalizing scales. ADHD symptoms were measured asking the mother to complete the ADHD Rating Scale-IV (ADHD-RS-IV) Home version for children at the 15-year assessment and the version for young adults at the 19-year assessment [42]. At 19 years, full IQ was obtained by a senior neuropsychologist with Wechsler adult intelligence scale, 3rd edition (WAIS-III) [43].

### Statistical analysis

Differences in cerebellar volume between the VLBW group and the control group were analyzed using a general linear model (GLM), adjusting for age, sex and eICV. For the non-normally distributed variables of psychiatric and perinatal data we used the Mann–Whitney U test. Differences in diagnostic levels were analyzed by the unconditional z-pooled test (http://www4.stat.ncsu.edu/~boos/exact/) [44].

All longitudinal changes in brain volumes were studied by means of mixed model linear regression, adjusting for sex and eICV. Mixed model methods allowed us to perform analyses combining cross-sectional and longitudinal data, accounting for missing data, irregular intervals between measures and within person dependence [45]. We investigated longitudinal differences in cerebellar WM and GM volumes between VLBW adolescents divided according to diagnostic status during adolescence (persisting/developing diagnosis vs. healthy/becoming healthy) and controls. We also analyzed the relationship between cerebellar WM and GM changes and the longitudinal changes of psychiatric symptoms and function assessed with questionnaires in the VLBW group (CGAS, ASEBA and ADHD-RS-IV).

We studied if there were cerebellar WM and GM volumetric differences between the two VLBW groups and the control group at 15 and 19 years of age by using a GLM, adjusting for age, sex and eICV. Linear regression was used to explore the relationship between cerebellar GM and WM volumes and psychiatric symptoms assessed with questionnaires at both 15 and 19 years of age, adjusting for age, sex and eICV. Normality of residuals was assessed by visual inspection of Q–Q plots. Missing cases were excluded pairwise. These analyses were further adjusted for IQ to elucidate the relationship between psychiatric diagnosis and symptoms, cognitive abilities and the cerebellum. However, results are presented before corrections to avoid shadowing the direct relationship between brain abnormalities and psychiatric symptoms [46].

Two-sided *p* values <0.05 were taken to indicate statistical significance, and 95 % confidence intervals (CI) are reported where relevant. All *p* values were corrected for multiple comparisons following the Benjamini-Hochberg procedure (50 comparisons) [47]. Data were analyzed using IBM SPSS Statistics versions 20 and 22 (SPSS, Chicago, IL) and STATA/IC 13.1 (Stata Corporation, College Station, TX, USA).

## Results

### Psychiatric and MRI findings

Neonatal and socio-demographic variables are displayed in Table 1. The VLBW group had lower IQ than the control group. There was no statistical significant difference in SES between the two groups. Cerebellar volumes and psychiatric outcome are given in Table 2. Compared with controls, the VLBW group had smaller volume of cerebellar WM at both ages, but cerebellar GM was not significantly different between the two groups at 15 or 19 years. The VLBW group had lower general psycho-social functioning expressed by lower CGAS scores at both ages. Psychiatric symptoms, measured by ASEBA-YSR and -ASR demonstrated no significant differences between the VLBW and the control group. The VLBW group had higher scores on the Inattention subscale of the ADHD-rating scale at 15 and 19 years. Compared with controls, there were more VLBW participants with psychiatric or subclinical diagnoses at both time points. In particular, the VLBW group had higher frequencies of ADHD diagnosis at both ages and higher frequency of anxiety disorders at 19 years.

**Table 1:** Participants’ neonatal and socio-demographic details

	Assessed at 15 years		Assessed 19 at years			Assessed at both time points	
	VLBW	Control	VLBW	Control		VLBW	Control
Number of participants	40	56	44	60		30	37
Males (%)	18 (45)	21 (37)	18 (41)	25 (42)		11 (37)	14 (38)
Background information							
Birthweight (grams) M (SD)	*1204* (236)***	3713 (500)	*1212* (234)***	3698 (501)		*1223* (250)***	3766 (544)
Gestational age (weeks)	*29.18* (2.65)***	39.61 (1.15)	*29.25* (2.54)***	39.72 (1.27)		*29.43* (2.60)***	39.51 (1.17)
Age (years-months) M (SD)	15–2 (0–6)	15–5 (0–5)	19–7 (0–7)	19–8 (0–6)	Time 1	15–2 (0–6)	15–5 (0–5)
					Time 2	19–9 (0–8)	19–7 (0–6)
IQ. M (SD)			*89.00* (12.54)***	99.85 (10.62)		*86.33* (13.52)***	100.14 (11.03)
SES (1–5). M (SD)	3.15 (1.25)	3.59 (1.04)	3.39 (1.38)	3.70 (0.95)		3.27 (1.33)	3.65 (0.92)

Linear regression adjusted for age and sex for normal distributed data, else the Mann–Whitney U-test

The unconditional z-pooled test was used to analyze differences in proportions between groups

*IQ* intelligence quotient, *M* mean, *SD* standard deviation, *SES* socio-economic status, *VLBW* very low birth weight (birth weight ≤ 1500)

* *p* ≤ 0.05, ** p ≤ 0.01, *** p ≤ 0.001 (VLBW versus controls)

**Table 2:** Cerebellar volume and psychiatric outcome in VLBW participants and controls

	Assessed at 15 years		Assessed 19 at years	
	VLBW (N = 40)	Control (N = 56)	VLBW (N = 44)	Control (N = 60)
Cerebellar volume (ml) M (SD)				
White matter M (SD)	*25.52* (4.05)*	28.93 (3.04)	*26.60* (4.03)*	29.83 (3.10)
Gray matter M (SD)	99.46 (11.02)	103.93 (10.08)	96.59 (11.14)	103.57 (8.85)
Psychiatric questionnaires				
*CGAS* M (SD)	*71.73* (14.48)***	86.96 (6.75)	*79.05* (12.75)**	85.78 (7.69)
ASEBA M (SD)				
Internalizing M (SD)	6.95 (5.27)	7.23 (5.96)	10.00 (9.45)	7.33 (7.25)
Externalizing M (SD)	7.68 (4.74)	8.14 (5.84)	7.25 (4.99)	6.48 (5.78)
Total problems. M (SD)	25.16 (14.91)	24.59 (15.81)	32.15 (21.53)	26.90 (20.78)
ADHD-RS-IV				
Hyperactivity M (SD)	2.78 (3.71)	1.43 (1.78)	2.90 (4.29)	1.34 (1.67)
Inattention M (SD)	*6.39* (5.11)***	2.51 (2.81)	*5.45* (5.58)**	1.76 (1.98)
Clinical diagnoses				
Any psychiatric diagnosis M (SD)	*12* (30)**	3 (5.36)	*11* (25)**	4 (6.67)
Anxiety disorders M (SD)	5 (12.50)	2 (3.57)	*7* (15.91)**	1 (1.67)
ADHD M (SD)	*3* (7.50)*	0 (0)	*4* (9.09)*	0 (0)
Other M (SD)	4 (10)	1 (1.76)	0 (0)	3 (5)
Any subclinical diagnosis n (%)	*11* (27.50)***	1 (1.76)	5 (11.36)	6 (10)
Anxiety n (%)	3 (7.50)	1 (1.76)	4 (9.09)	2 (3.33)
ADHD n (%)	*8* (20)***	0 (0)	1 (2.27)	3 (5)
Other n (%)	0 (0)	0 (0)	0 (0)	1 (1.67)
Diagnostic status n (%)				
Healthy/became healthy n (%)	*22* (*55*)**	46 (82)	*25* (*57*)**	50 (83)
Persisting/developed diagnosis n (%)	*18* (*45*)**	10 (18)	*16* (*36*)*	9 (15)

Linear regression adjusted for age and sex for normal distributed data, else the Mann–Whitney U-test. Cerebellar volumes adjusted for estimated intracranial volume. The unconditional z-pooled test was used to analyze differences in proportions between groups

*ADHD-RS-IV* attention-deficit/hyperactivity disorder rating scale, *ASEBA* the Achenbach system of empirically based assessment, *YSR* (Youth Self Report at 14 years) and *ARS* (Adult Self Report at 19 years), *CGAS* children’s global assessment scale, *M* mean, *SD* standard deviation, *VLBW* very low birth weight (birth weight ≤ 1500)

* p ≤ 0.05, ** p ≤ 0.01, *** *p* ≤ 0.001 (VLBW versus controls)

### Relationship between clinical and MRI data

#### Cerebellar growth rate and psychiatric data

**Fig. 2:**
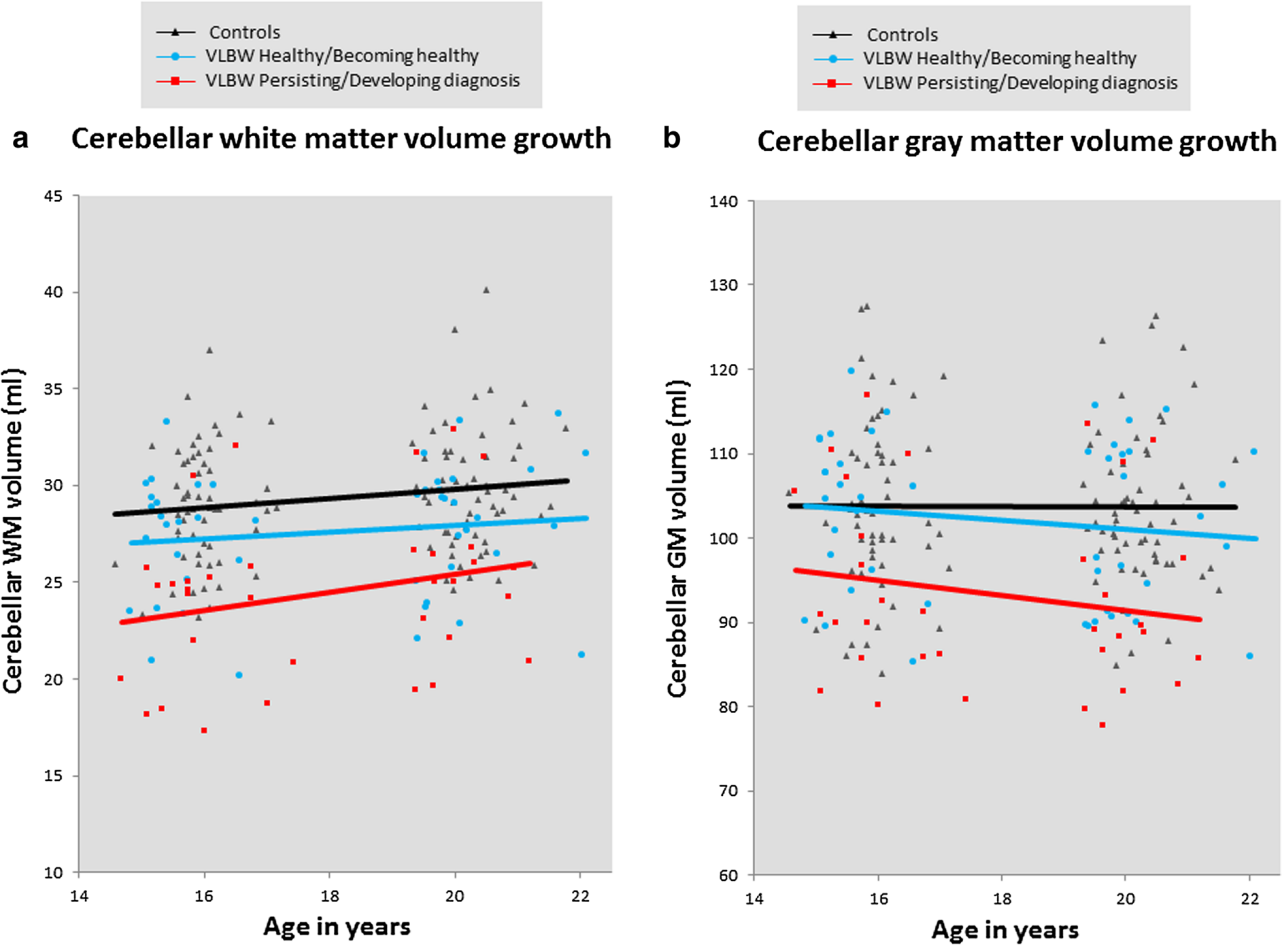
Cerebellar volume change in VLBW adolescents according to diagnostic group and controls. Cerebellar WM (**a**) and GM (**b**) volume change during adolescence was similar for the two VLBW groups and controls. *GM* gray matter, *Ml* milliliters, *VLBW* very low birthweight, *WM* white matter

Mixed model linear regression results for the differences in cerebellar growth rate from 15 to 19 years of age between the two VLBW groups and controls are provided in Table 3. We did not find any differences in cerebellar growth between the two VLBW groups and controls (Fig. 2).

**Table 3:** Cerebellar growth differences between the two VLBW groups and the control group from 15 to 19 years of age

	Interaction time × group		
	Coefficient	(95 % CI)	*p* *value*
Cerebellar white matter	−0.115	(−0.410 to 0.181)	0.447
Cerebellar gray matter	0.395	(−0.226 to 1.015)	0.213

Mixed linear regressions with groups of severity of diagnosis and time as independent variables and brain volumes (ml) as dependent variable. Adjusted for sex and estimated intracranial volume, but not for IQ

*CI* confidence interval, *IQ* intelligence quotient, *VLBW* very low birth weight

Mixed model linear regression analyses in the VLBW group between cerebellar volumetric changes over time and the development of psychiatric symptoms assessed with questionnaires (Additional file 1: Appendix S1) revealed an association between cerebellar WM volume increases over time and higher ADHD-RS-IV Inattention scores [B = 0.621 (0.0629–1.180) *p* = 0.029]. However, this association disappeared after corrections for multiple comparisons.

#### Cerebellar volumes and severity of diagnosis

**Fig. 3:**
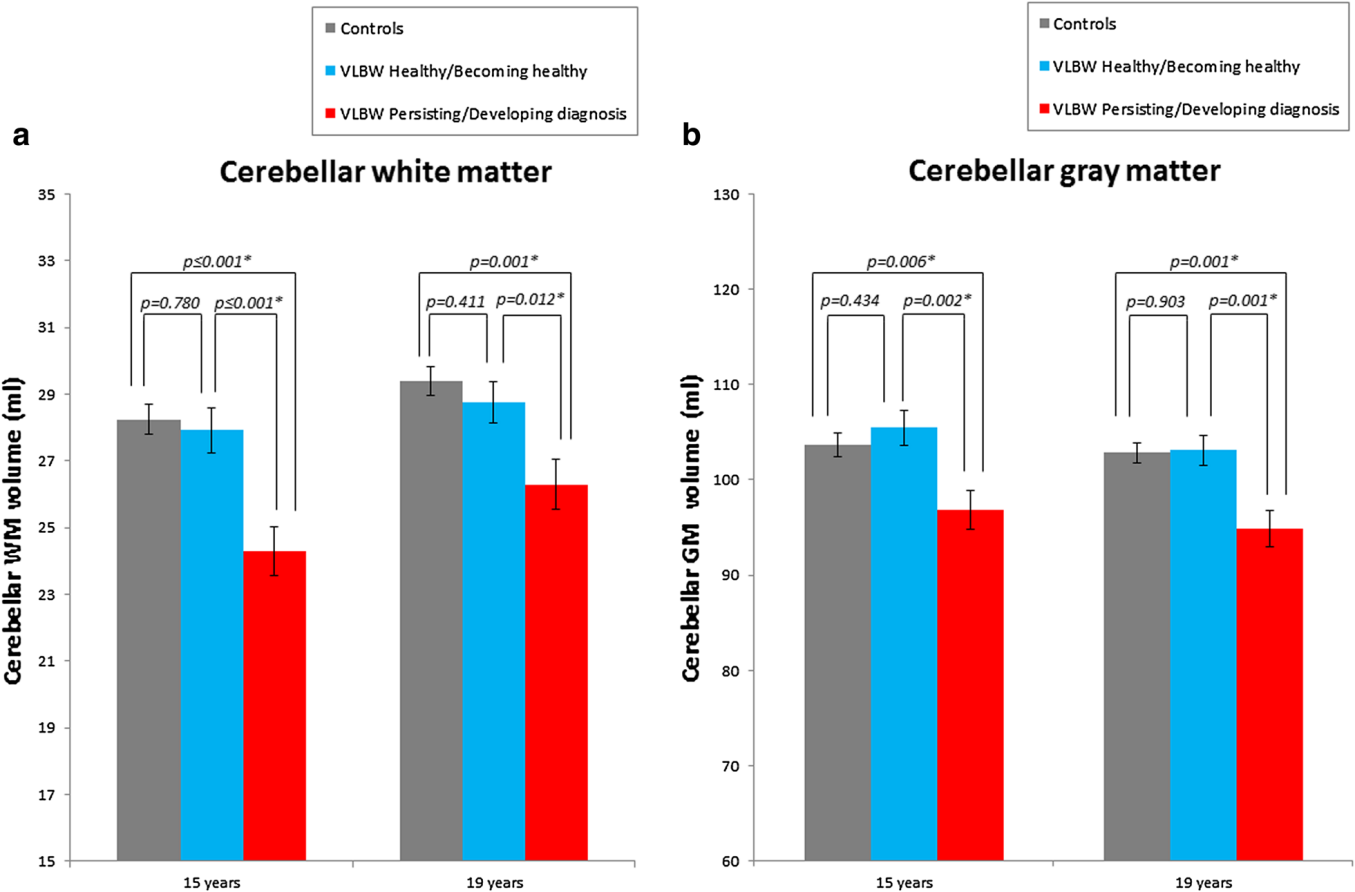
Cerebellar WM (**a**) and GM (**b**) volumes at 15 and 19 years of age in the two VLBW groups of diagnostic group and controls. Mean cerebellar volumes adjusted for age, sex and estimated intracranial volume. The *asterisks* (*) indicate significant results after adjusting for multiple testing. *GM* gray matter, *Ml* milliliters, *VLBW* very low birthweight, *WM* white matter

Comparisons between cerebellar volumes in the two VLBW groups and controls are presented in Fig. 3. At both 15 and 19 years of age, VLBW adolescents who had or developed a psychiatric diagnosis during adolescence had smaller cerebellar WM and GM volumes than controls. This VLBW group had also smaller cerebellar WM and GM volumes than VLBW adolescents who were or became healthy in this period. After correcting for multiple comparisons, all results remained significant. Significance also remained after adjusting for IQ, except for cerebellar GM differences at 15 years. Detailed results before and after correction for IQ are provided in Additional file 2: Appendix S2A and B, respectively.

#### Cerebellar volumes and psychiatric symptoms assessed with questionnaires

**Fig. 4:**
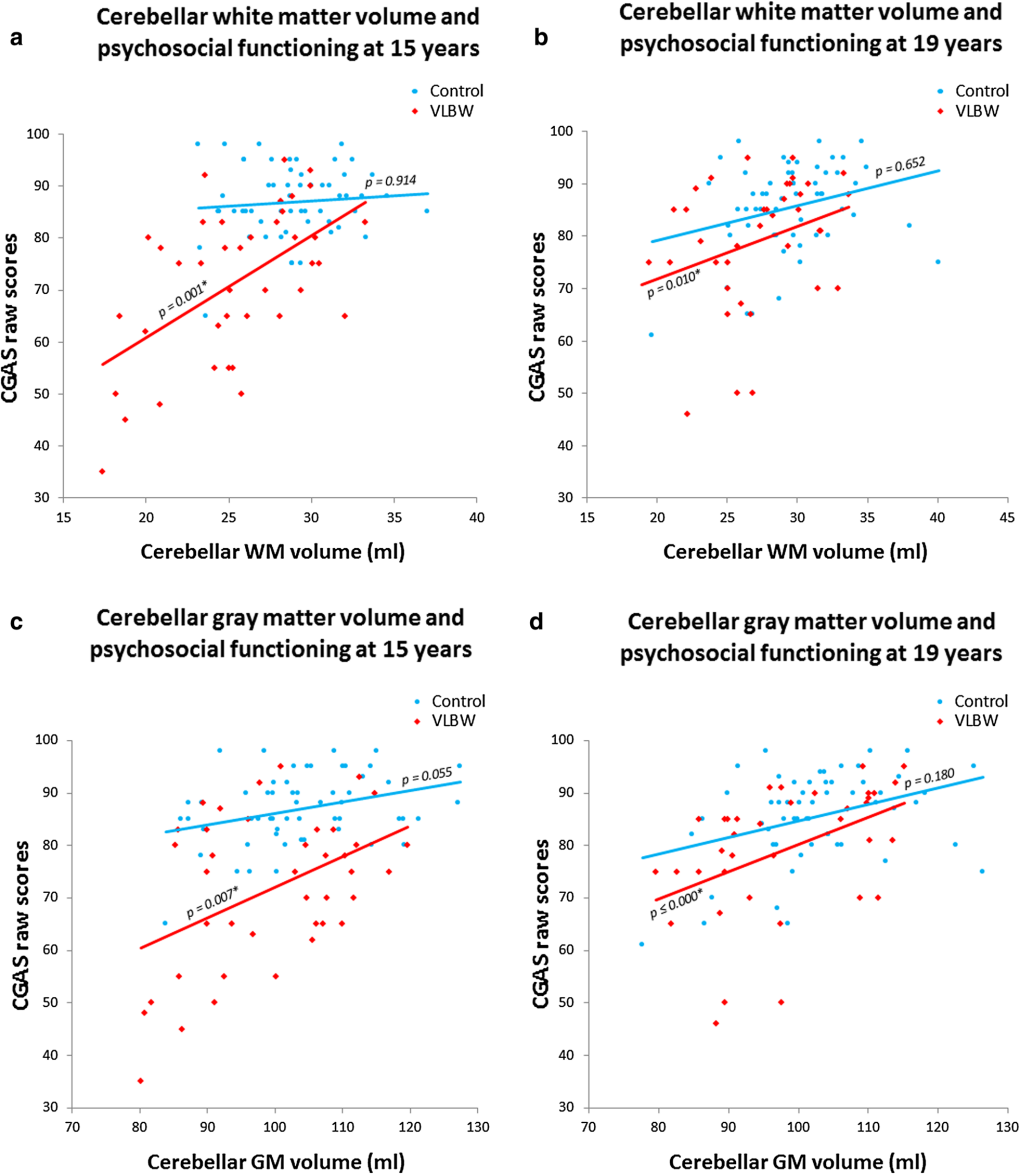
Cerebellar volumes and psychosocial functioning at 15 and 19 years of age. The *top panels* show cerebellar WM volume and psychosocial functioning at 15 (**a**) and 19 (**b**) years. The *bottom panels* depict cerebellar GM volume and psychosocial functioning at 15 (**c**) and 19 (**d**) years. Absolute cerebellar volumes. The *asterisks* (*) indicate significant results after adjusting for multiple testing. *Ml* milliliters, *VLBW* very low birth weight, *CGAS* children’s global assessment scale, *GM* gray matter, *WM* white matter

**Fig. 5:**
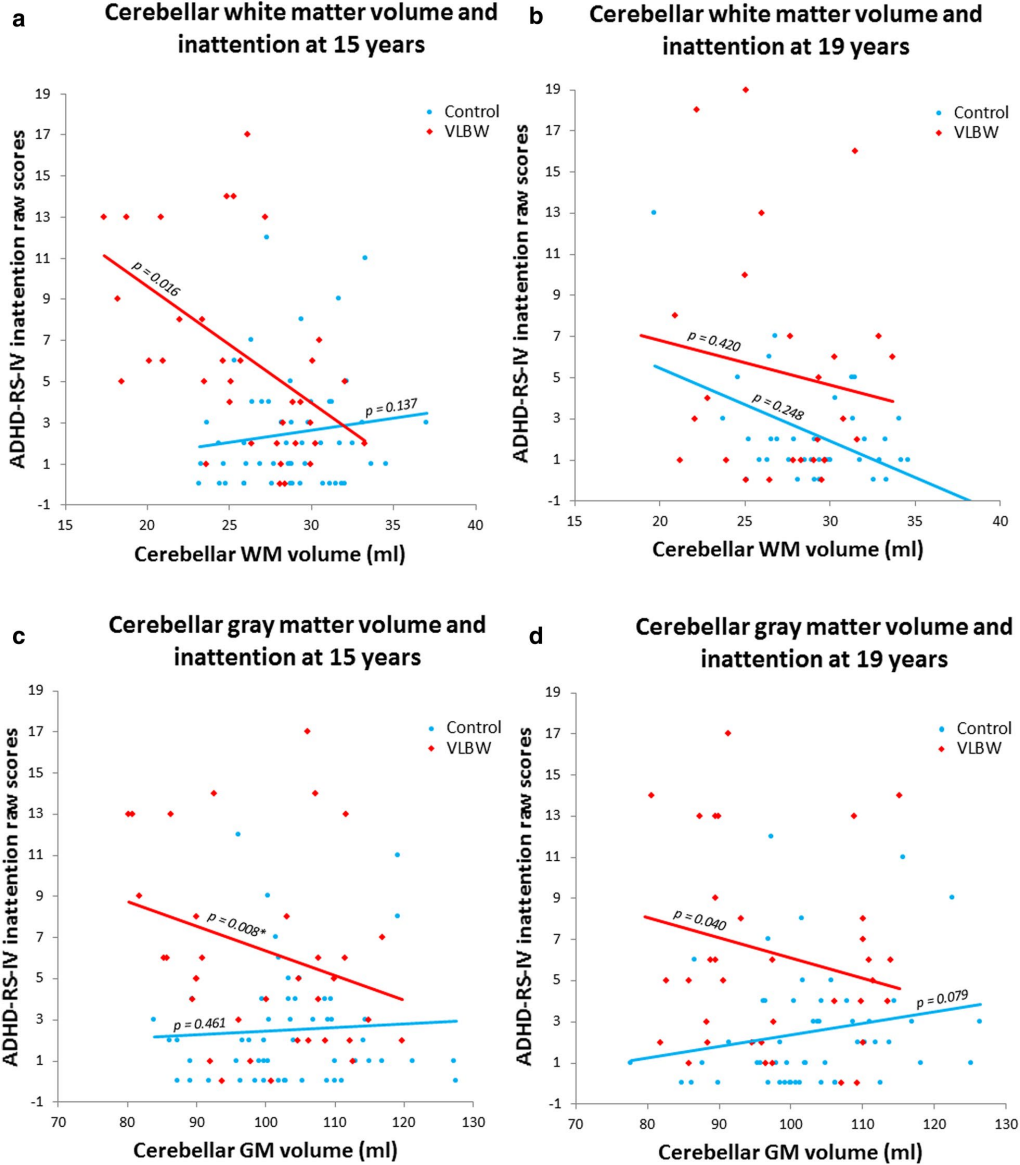
Cerebellar volumes and inattention at 15 and 19 years of age. The *top panels* show cerebellar WM volume and inattention at 15 (**a**) and 19 (**b**) years. The *bottom panels* depict cerebellar GM volume and inattention at 15 (**c**) and 19 (**d**) years. Absolute cerebellar volumes. The *asterisks* (*) indicate significant results after adjusting for multiple testing. *ADHD-RS-IV* ADHD-rating scale-IV, *Ml* milliliters, *VLBW* very low birth weight, *CGAS* children’s global assessment scale, *GM* gray matter, *WM* White matter

Linear regression results between cerebellar volumes and psychiatric symptoms and function assessed by questionnaires in the VLBW group are provided in Table 4. Lower CGAS scores, indicating lower psychosocial functioning, were associated with smaller cerebellar WM and GM volumes at both 15 and 19 years of age (Fig. 4). These results remained significant after correcting for multiple comparisons. We did not find any associations between cerebellar volumes and the ASEBA composite scales, nor with ADHD-RS-IV Hyperactivity scores. However, we found that the ADHD-RS-IV Inattention scores were associated with smaller cerebellar GM volumes at both ages and with smaller WM volume at 15 years (Fig. 5). After correcting for multiple testing, significance only remained for cerebellar GM differences at 15 years of age (Fig. 5c). When we further corrected the analyses for IQ, only significant differences between smaller cerebellar GM volumes and poorer psychosocial functioning (CGAS) at 19 years remained. Results with IQ corrections are provided in Additional file 3: Appendix S3.

**Table 4:** Linear regression with psychiatric data as dependent variable and cerebellar volumes (ml) as independent variable in the VLBW group

	15 years			19 years		
	Coefficient	(95 % CI)	*p* value	Coefficient	(95 % CI)	*p* *value*
CGAS (15 years n = 40, 19 years n = 41)						
Cerebellar WM	1.930	(0.841 to 3.020)	*0.001* ^a^	1.450	(0.362 to 2.539)	*0.010* ^a^
Cerebellar GM	0.630	(0.187 to 1.072)	*0.007* ^a^	0.764	(0.382 to 1.147)	*<0.000* ^a^
ASEBA (15 years n = 38, 19 years n = 40)						
Internalizing						
Cerebellar WM	−0.259	(−0.735 to 0.217)	0.276	−0.454	(−1.317 to 0.410)	0.294
Cerebellar GM	−0.118	(−0.301 to 0.064)	0.196	−0.287	(−0.611 to 0.038)	0.082
Externalizing						
Cerebellar WM	−0.098	(−0.516 to 0.320)	0.637	−0.223	(−0.70 to 0.254)	0.349
Cerebellar GM	−0.130	(−0.285 to 0.026)	0.099	−0.099	(−0.283 to 0.085)	0.282
Total problems						
Cerebellar WM	−0.818	(−2.142 to 0.506)	0.217	−1.190	(−3.201 to 0.820)	0.238
Cerebellar GM	−0.457	(−0.955 to 0.040)	0.070	−0.683	(−1.441 to 0.074)	0.075
ADHD-RS-IV (15 years n = 36, 19 years n = 29)						
Hyperactivity						
Cerebellar WM	−0.286	(−0.597 to 0.026)	0.071	0.158	(−0.348 to 0.663)	0.526
Cerebellar GM	−0.092	(−0.214 to 0.030)	0.135	0.007	(−0.190 to 0.204)	0.940
Inattention						
Cerebellar WM	−0.528	(−0.950 to 0.105)	*0.016*	−0.256	(−0.899 to 0.387)	0.420
Cerebellar GM	−0.222	(−0.382 to 0.061)	*0.008* *	−0.243	(−0.473 to 0.012)	*0.040*

Adjusted for age, sex and estimated intracranial volume, but not for IQ

*ADHD-RS-IV* attention-deficit/hyperactivity disorder rating scale, *ASEBA* Achenbach system of empirically based assessment, *YSR* (Youth Self Report at 14 years) and *ARS* (Adult Self Report at 19 years), *CGAS* children’s global assessment scale, *CI* confidence interval, *GM* gray matter, *IQ* intelligence quotient, *VLBW* very low birth weight, *WM* white matter

^**a**^Significant results also when corrected for multiple comparisons using the Benjamini–Hochberg procedure

## Discussion

We studied the relationship between cerebellar volumes and psychiatric symptoms and diagnoses at 15 and 19 years of age in adolescents born very preterm and with VLBW. Cerebellar growth trajectories from 15 to 19 years of age were equal between adolescents born with VLBW and controls, regardless of psychiatric morbidity (Fig. 2). However, VLBW adolescents with a persisting/developing diagnosis during adolescence had maintained smaller cerebellar WM and GM volumes compared with controls and compared with VLBW adolescents who were healthy or became healthy during this period. Moreover, cerebellar volumes did not differ between VLBW adolescents who were or became healthy from 15 to 19 years of age and controls (Fig. 3). At both 15 and 19 years of age, larger cerebellar WM and GM volumes correlated with better general psychosocial functioning in the VLBW group (Fig. 4).

Smaller cerebellar volumes have been consistently reported in preterm children compared with term-born peers from birth [6, 48–51] to childhood [52] and adolescence [27–29, 53–55]. Even though overall smaller brain volumes are a common trait in children born very preterm and with VLBW [55], some studies suggest that there are not brain growth differences, including the cerebellum, between individuals born preterm and term-born peers [29, 55, 56]. Nonetheless, other studies have found differences in cerebellar trajectories between preterm and term-born children during adolescence. In an MRI longitudinal study, Parker et al. [27] reported total cerebellar volume reduction from 15 to 18 years of age in a cohort of adolescents born very preterm compared with term-born peers. This reduction in cerebellar volume was associated with having more problems in several questionnaire items concerning concentration, feeling useful, decision-making capability, overcoming difficulties, feeling confident and feeling worthless. Abnormal cerebellar growth has also been reported to occur right after birth, even after normal cerebellar ultrasound [6, 48]. Preterm children with the most deviant cerebellar development have higher rates of intraventricular hemorrhage and other associated complications like post-hemorrhagic hydrocephalus and neurosurgical interventions [50, 57–59]. However, extreme prematurity has been noted as the most explicative factor for disruptive cerebellar development in VLBW neonates [6, 51, 57, 59, 60]. The causes of deviant cerebellar development in the absence of apparent damage are unknown [9]. We speculate that the smaller cerebellar volumes in VLBW adolescents with persistent/increasing mental health problems might be originated in the perinatal/neonatal period.

In order to properly understand the role of the cerebellum in the appearance and maintenance of psychiatric disorders in children born very preterm and with VLBW, it is important to study its anatomy and how premature birth affects its development. The cerebellum is connected with the whole brain, especially with the cerebral cortex [24, 61, 62]. The vast development of the cerebellum occurs, mainly, in the third trimester of gestation, where the cerebellum increases its volume fourfold [6]. Early disruption of the cerebellar circuitry development has been positively correlated with ASD, attention deficit and emotional problems [25]. In fact, a meta-analysis pointed out cerebellar abnormalities as the most consistently reported structural finding for ADHD [63], but the results whether total cerebellar volume, WM or GM or both are abnormal were inconclusive. ADHD symptom severity has been shown to correlate with overall cerebellar volume [64]. Many studies have found cerebellar abnormalities in ADHD, especially in the vermis [65–71]. Our finding of smaller cerebellar GM volume associated with higher inattention symptoms in the VLBW group points out GM as related to their symptoms. However, the lack of correlation with WM volumes might be due to low statistical power. Diffusion tensor imaging studies investigating WM differences in ADHD have reported reduced fractional anisotropy in the middle cerebellar peduncles [72, 73], and WM in the left cerebellum [73, 74]. A recent fMRI study has reported a link of abnormal cerebro-cerebellar connectivity with ADHD, and particularly for inattention, suggesting that ADHD symptoms are not the direct result of cerebellar abnormalities, but the result of damages in the whole network [75].

Injury confined to the cerebellum has been associated with reduced growth in specific regions of the uninjured contralateral cerebral cortex in preterm born neonates (dorsolateral prefrontal, premotor, sensorimotor, and mid-temporal regions), and this secondary growth reduction has been associated with a higher risk of impaired cognitive, language, behavior, and motor performance [49, 76]. This suggests that injury to the immature cerebellum could affect neurologic function through mechanisms that interfere with later development of remote regions of the cerebral cortex [26]. Projections from the cerebellum to the cerebral cortex constitute the cerebello-thalamo-cortical (CTC) pathway, the main efferent cerebellar projection [23, 24]. The CTC pathway has been linked with deficits in information processing in schizophrenia [77, 78], and disruption in cortico-cerebellar connectivity has been proposed as a major neurobiological mechanism of emotional dysregulation [79]. In preterm infants, thalamo-cortical projections measured with DTI were affected compared with born-at-term controls, suggesting that such pathways might be especially vulnerable to preterm birth [80]. Different fMRI studies have also shown activation of the cerebellum in different emotional tasks, such us viewing pictures of deceased loved ones [81] and viewing pictures and movies evoking both negative and positive feelings [82, 83]. These results suggest that the cerebellum is directly involved in the appraisal of emotional stimuli. The closed-loop circuits between the cerebellum and the cerebral cortex serve as the anatomical substrate by which the cerebellum can modulate activation patterns in distal regions. Based on these interactions, it has been proposed that cerebellar dysfunction or disruption early in development could have major impacts on the structure and function of the cortical regions to which it projects [2]. We hypothesize that deviant cerebellar development, probably occurring around the neonatal period, might cause smaller cerebellar volumes that are carried onto adolescence affecting the cerebellar circuitry and its extensive connections with the brain, and that this injury pattern probably plays an important role in the development and maintenance of psychiatric disorders in this population. Moreover, discriminating VLBW children with significant smaller cerebellar volumes than controls might help to identify which of them are at risk of developing psychiatric problems, and it might aid to take preventive actions at an earlier stage.

In order to clarify the relationship between psychiatric symptoms, general cognitive abilities and cerebellar volumes, we corrected our analyses for IQ. After this, all the results remained significant, except for differences in cerebellar GM volume at 15, the relationship between cerebellar volumes and inattention, and cerebellar WM volumes and psychosocial function at both ages and GM at 15. IQ summarizes psychological processes such as attention, executive functions, and general knowledge [84], while social cognition measures are not properly covered. Having intellectual disability has been linked to higher prevalence of psychiatric disorders [85], and recent research suggests that impaired executive functions (i.e. inhibition, working memory, and cognitive flexibility) is a core feature in many mental illnesses. If a deficit in executive functions is a core symptom of psychiatric disorders, correcting for IQ might be an overcorrection in brain imaging studies trying to find the neural basis of mental health illness [86]. Still, the nature of this correlation is not fully understood yet [85, 87]. A cohort study using data from over 900,000 individuals in Sweden has linked higher IQ scores during adolescence with higher risk of developing bipolar disorder later in life, controlling for socioeconomic group and parental education [88]. At the same time, neurocognitive deficits in bipolar disorder have been highlighted as an important determinant of the disruptive nature of this disorder [89]. In preterm born individuals, cognitive abilities might be affected by the same brain mechanisms that affect mental health problems. Thus, if IQ, which is also the result of brain characteristics [90], is a mediating variable in mental health disorders, rather than a core feature, correcting for IQ might shadow the direct relationship between brain abnormalities and psychiatric symptoms [46]. More research is needed to clarify this matter.

The strength of this study is the usage of both questionnaires and a semi-structured diagnostic interview for identifying psychiatric symptoms and disorders, thereby contributing to a comprehensive psychiatric evaluation. Another strength is the combined cross-sectional and longitudinal design with clinical and MRI assessments at both 15 and 19 years of age in this cohort. The participation rate was comparable to other follow-up studies with similar study groups [91] and participants and non-participants did not differ in perinatal variables (gestational age, birth weight, maternal age at birth), making selection bias less likely. Another strength is that the groups did not differ in levels of socio-economic status.

We used a well-known and reliable automated MR segmentation method to measure cerebellar volumes (http://surfer.nmr.mgh.harvard.edu/). However, this method only provides delineation of the total cerebellum, differentiating between WM and GM, but not accounting for the cerebellar vermis. FreeSurfer has been shown to underestimate the cerebellar region [92] and problems to distinguish perfectly between the cerebellum and brain stem [93]. We inspected all processed images for accuracy of the FreeSurfer segmentation and rejected structures with obvious segmentation errors. However, in order to avoid introducing bias and increasing variances into the data set of MRI images, no manual editing was performed. Further studies using MRI with higher field strength or improved algorithms for segmentation that allows more detailed cerebellar segmentations, may be required to support or reject our results.

Another limitation of the study is group size. Due to the relatively small sample, only large differences and strong associations could reach significant levels. Hence, negative findings should be interpreted with caution. We had longitudinal data for a smaller sample than the cross-sectional study groups, which reduced the statistical power and hence, the generalization of the longitudinal results. Studies with larger samples are certainly needed to confirm the findings.

The ASEBA self-report composite scales were not associated with cerebellar volumes, while results from diagnostic evaluation were. This can be explained by a discrepancy in our VLBW group between the ASEBA self-report and the clinical reports at both assessments, suggesting that the VLBW group might be under-reporting problems on questionnaires [4, 94]. Similar discrepancies have been reported in other studies between self-reports and parent-reports in VLBW young adult populations [95, 96]. Therefore, the lack of association with the ASEBA subscales should be interpreted with this in mind.

## Conclusion

Cerebellar growth rate was the same for VLBW children and term-born peers regardless of psychiatric status. Still, VLBW participants who had persistent or developed psychiatric problems during adolescence presented smaller cerebellar WM and GM volumes compared with controls, and also compared with VLBW adolescents who were or became healthy during this period. Our results suggest that significant smaller cerebellar volumes may be a biomarker for developing and maintaining psychiatric problems during adolescence in individuals born with VLBW.

## Additional files

10.1186/s13034-016-0093-8 Mixed model linear regressions with psychiatric data as dependent variable and cerebellar volumes (ml) and time as independent variables in the VLBW group. Adjusted for age, gender and estimated intracranial volume, but not for IQ.
10.1186/s13034-016-0093-8 Cerebellar volume (ml) differences between the two VLBW diagnostic groups and controls at 15 and 19 years of age. B. Cerebellar volume (ml) differences between the two VLBW diagnostic groups and controls at 15 and 19 years of age adjusted for IQ.
10.1186/s13034-016-0093-8 Linear regression with psychiatric data as dependent variable and cerebellar volumes (ml) as independent variable in the VLBW group adjusted for IQ.

## Abbreviations

ASEBA: Achenbach system of empirically based assessment
ADHD-RS-IV: ADHD rating scale-IV
CGAS: children’s global assessment scale
CTC: cerebello-thalamo-cortical
GM: gray matter
VLBW: very low birth weight
WM: white matter
