# Supplementary material for: Mental health and cerebellar volume during adolescence in very-low-birth-weight infants: a longitudinal study
Source: Child Adolesc Psychiatry Ment Health. 2016 Mar 16;10:6. doi: 10.1186/s13034-016-0093-8 (PMC4793750; doi:10.1186/s13034-016-0093-8)
Supplement: Supplementary file 1 — 10.1186/s13034-016-0093-8 Mixed model linear regressions with psychiatric data as dependent variable and cerebellar volumes (ml) and time as independent variables in the VLBW group. Adjusted for age, gender and estimated intracranial volume, but not for IQ. [file 13034_2016_93_MOESM1_ESM.docx]

| **Appendix S1:**  Mixed model linear regressions with psychiatric data as dependent variable and cerebellar volumes (ml) and time as independent variables in the VLBW group. Adjusted for age, gender and estimated intracranial volume, but not for IQ. | | | |
| --- | --- | --- | --- |
|  | **Interaction time x cerebellum** | | |
|  | ***Coefficient*** | ***(95% ci)*** | ***p-value*** |
| ***CGAS*** *(15 years n*=*40, 19 years n=41)* |  |  |  |
| Cerebellar white matter | -0.283 | ( -1.251 to 0.687) | 0.568 |
| Cerebellar gray matter | 0.145 | (-0.222 to 0.512) | 0.439 |
| **ASEBA self-report** *(15 years n*=38*, 19 years n=40)* | | | |
| **Internalizing** |  |  |  |
| Cerebellar white matter | -0.349 | (-1.110 to 0.414) | 0.371 |
| Cerebellar gray matter | -0.178 | (-0.444 to 0.0893) | 0.192 |
| **Externalizing** |  |  |  |
| Cerebellar white matter | - 0.170 | (-0.668 to 0.328) | 0.502 |
| Cerebellar gray matter | 0.010 | (-0.172 to 0.192) | 0.913 |
| **Total problems** |  |  |  |
| Cerebellar white matter | -0.768 | (-2.698 to 1.163) | 0.436 |
| Cerebellar gray matter | -0.289 | (-0.975 to 0.395) | 0.407 |
| **ADHD-RS mother-report** *(15 years n*=36, *19 years n=29)* | | | |
| **Hyperactivity** |  |  |  |
| Cerebellar white matter | -0.105 | (-0.583 to 0.374) | 0.668 |
| Cerebellar gray matter | -0.153 | (-0.319 to 0.0139) | 0.072 |
| **Inattention** |  |  |  |
| Cerebellar white matter | 0.621 | (0.0629 to 1.180) | **0.029** |
| Cerebellar gray matter | 0.100 | ( -0.104 to 0.303) | 0.336 |
| *Abbreviations*: ADHD-RS: Attention Deficit Hyperactivity Disorder Rating Scale; ASEBA: Achenbach System of Empirically Based Assessment, YSR (Youth Self Report at 14 years) and ARS (Adult Self Report at 19 years); CGAS: Children’s Global Assessment Scale; IQ: Intelligence Quotient; Ml: Milliliters; VLBW: Very low birth weight. | | | |
