# Supplementary material for: Mental health and cerebellar volume during adolescence in very-low-birth-weight infants: a longitudinal study
Source: Child Adolesc Psychiatry Ment Health. 2016 Mar 16;10:6. doi: 10.1186/s13034-016-0093-8 (PMC4793750; doi:10.1186/s13034-016-0093-8)
Supplement: Supplementary file 2 — 10.1186/s13034-016-0093-8 Cerebellar volume (ml) differences between the two VLBW diagnostic groups and controls at 15 and 19 years of age. B. Cerebellar volume (ml) differences between the two VLBW diagnostic groups and controls at 15 and 19 years of age adjusted for IQ. [file 13034_2016_93_MOESM2_ESM.docx]

| **Appendix S2A.** Cerebellar volume (ml) differences between the two VLBW diagnostic groups and controls at 15 and 19 years of age. | | | | | | | | | | | |
| --- | --- | --- | --- | --- | --- | --- | --- | --- | --- | --- | --- |
|  | | **MD** | **SE** | | **(95% ci)** | ***p*-value** | **MD** | **SE** | **(95% ci)** | | ***p*-value** |
|  | | **15 years** | | | | | **19 years** | | | | |
| **Cerebellar white matter** | |  |  |  | |  |  |  |  |  | |
| Increasing/Developing diagnosis | Healthy/Becoming healthy | -3.726 | 0.988 | (-5.689 to -1.763) | | **≤0.001*** | -2.458 | 0.961 | (-4.365 to -0.551) | **0.012*** | |
|  | Controls | -4.023 | 0.896 | (-5.804 to -2.243) | | **≤0.001*** | -3.109 | 0.888 | (-4.872 to -1.345) | **0.001*** | |
| Healthy/Becoming healthy | Controls | -0.297 | 0.862 | (-2.009 to 1.414) | | 0.731 | -0.651 | 0.789 | (-2.216 to 0.915) | 0.411 | |
| **Cerebellar gray matter** | |  |  |  | |  |  |  |  |  | |
| Increasing/Developing diagnosis | Healthy/Becoming healthy | -8.000 | 2.653 | (-13.271 to -2.729) | | **0.003*** | -8.228 | 2.406 | (-13.005 to -3.452) | **0.001*** | |
|  | Controls | -7.000 | 2.407 | (-11.783 to -2.220) | | **0.005*** | -7.986 | 2.225 | (-12.404 to -3.568) | **0.001*** | |
| Healthy/Becoming healthy | Controls | 0.999 | 2.313 | (-3.598 to 5.595) | | 0.667 | 0.242 | 1.975 | (-3.679 to 4.163) | 0.903 | |
| General Linear Model brain volumes (ml) as dependent variable and group as categorical independent variable. Adjusted for age, sex and estimated intracranial volume, but not IQ.  ***** Significant results corrected for multiple comparisons using the Benjamini-Hochberg procedure.  *Abbreviations*: ci: Confidence interval; IQ: Intelligence Quotient; MD: Mean difference; Ml: Milliliters; SE: Standard error; VLBW: Very low birth weight. | | | | | | | | | | | |

| **Appendix S2B.** Cerebellar volume (ml) differences between the two VLBW diagnostic groups and controls at 15 and 19 years of age adjusted for IQ | | | | | | | | | | |
| --- | --- | --- | --- | --- | --- | --- | --- | --- | --- | --- |
|  | | **MD** | **SE** | **(95% ci)** | ***p*-value** | **MD** | **SE** | **(95% ci)** | | ***p*-value** |
|  | | **15 years** | | | | **19 years** | | | | |
| **Cerebellar white matter** | |  |  |  |  |  |  |  |  | |
| Increasing/Developing diagnosis | Healthy/Becoming healthy | -2.632 | 1.043 | (-4.705 to -0.560) | **0.013** | -2.317 | 0.989 | (-4.281 to -0.353) | **0.021** | |
|  | Controls | -2.544 | 1.016 | (-4.564 to -0.524) | **0.014** | -2.864 | 0.971 | (-4.793 to -0.936) | **0.004*** | |
| Healthy/Becoming healthy | Controls | 0.089 | 0.839 | (-1.578 to 1.755) | 0.916 | -0.547 | 0.808 | (-2.151 to 1.056) | 0.500 | |
| **Cerebellar gray matter** | |  |  |  |  |  |  |  |  | |
| Increasing/Developing diagnosis | Healthy/Becoming healthy | -5.611 | 2.873 | (-11.320 to 0.097) | **0.054** | -7.768 | 2.473 | (-12.680 to -2.857) | **0.002*** | |
|  | Controls | -3.925 | 2.799 | (-9.488 to 1.638) | 0.164 | -7.190 | 2.429 | (-12.013 to -2.367) | **0.004*** | |
| Healthy/Becoming healthy | Controls | 1.686 | 2.310 | (-2.905 to 6.277) | 0.467 | 0.579 | 2.020 | (-3.432 to 4.589) | 0.775 | |
| General Linear Model brain volumes (ml) as dependent variable and group as categorical independent variable. Adjusted for age, sex, estimated intracranial volume and IQ.  ***** Significant results corrected for multiple comparisons using the Benjamini-Hochberg procedure.  *Abbreviations*: ci: Confidence interval; IQ: Intelligence Quotient; MD: Mean difference; Ml: Milliliters; SE: Standard error; VLBW: Very low birth weight. | | | | | | | | | | |
