# Supplementary material for: Mental health and cerebellar volume during adolescence in very-low-birth-weight infants: a longitudinal study
Source: Child Adolesc Psychiatry Ment Health. 2016 Mar 16;10:6. doi: 10.1186/s13034-016-0093-8 (PMC4793750; doi:10.1186/s13034-016-0093-8)
Supplement: Supplementary file 3 — 10.1186/s13034-016-0093-8 Linear regression with psychiatric data as dependent variable and cerebellar volumes (ml) as independent variable in the VLBW group adjusted for IQ. [file 13034_2016_93_MOESM3_ESM.docx]

| **Appendix S3.**  Linear regression with psychiatric data as dependent variable and cerebellar volumes (ml) as independent variable in the VLBW group adjusted for IQ. | | | | | | |
| --- | --- | --- | --- | --- | --- | --- |
|  | **15 years** | | | **19 years** | | |
|  | ***Coefficient*** | ***(95% CI)*** | ***p-value*** | ***Coefficient*** | ***(95% CI)*** | ***p-value*** |
| ***CGAS*** *(15 years n*=*40, 19 years n=41)* |  |  |  |  |  |  |
| Cerebellar WM | 0.486 | (-0.898 to 1.869) | 0.476 | 0.747 | (-0.399 to 1.894) | 0.194 |
| Cerebellar GM | 0.038 | (-0.467 to 0.546) | 0.878 | 0.585 | (0.206 to 0.963) | **0.003*** |
| **ASEBA** *(15 years n*=38*, 19 years n=40)* | |  |  |  |  |  |
| **Internalizing** |  |  |  |  |  |  |
| Cerebellar WM | -0.168 | (-0.911 to 0.574) | 0.709 | -0.280 | (-1.268 to 0.708) | 0.569 |
| Cerebellar GM | -0.080 | (-0.347 to 0.188) | 0.543 | -0.249 | (-0.601 to 0.102) | 0.158 |
| **Externalizing** |  |  |  |  |  |  |
| Cerebellar WM | 0.013 | (-0.398 to 0.424) | 0.949 | -0.223 | (-0.701 to 0.2549 | 0.349 |
| Cerebellar GM | -0.063 | (-0.231 to 0.105) | 0.448 | -0.099 | (-0.283 to 0.085) | 0.282 |
| **Total problems** |  |  |  |  |  |  |
| Cerebellar WM | -0.149 | (-2.133 to 1.834) | 0.878 | -0.691 | (-2.981 to 1.598) | 0.544 |
| Cerebellar GM | -0.195 | (-0.907 to 0.518) | 0.577 | -0.565 | (-1.381 to 0.251) | 0.168 |
| **ADHD-RS-IV** *(15 years n*=36, *19 years n=29)* | |  |  |  |  |  |
| **Hyperactivity** |  |  |  |  |  |  |
| Cerebellar WM | 0.171 | (-0.627 to 0.284) | 0.444 | 0.124 | (-0.539 to 0.787) | 0.702 |
| Cerebellar GM | -0.013 | (-0.180 to 0.153) | 0.870 | -0.160 | (-0.393 to 0.072) | 0.167 |
| **Inattention** |  |  |  |  |  |  |
| Cerebellar WM | -0.227 | (-0.806 to 0.352) | 0.425 | 0.281 | (-0.296 to 0.857) | 0.324 |
| Cerebellar GM | -0.095 | (-0.303 to 0.113) | 0.356 | 0.029 | (-0.186 to 0.243) | 0.786 |
| Adjusted for age, sex, estimated intracranial volume and IQ.  * Significant results corrected for multiple comparisons using the Benjamini-Hochberg procedure.  *Abbreviations*: ADHD-RS-IV: Attention-Deficit/Hyperactivity Disorder Rating Scale; ASEBA: Achenbach System of Empirically Based Assessment, (Youth Self Report at 14 years) and ARS (Adult Self Report at 19 years); CGAS: Children’s Global Assessment Scale; CI: Confidence interval; GM: Gray matter; IQ: Intelligence Quotient; Ml: Milliliters; VLBW: Very low birth weight; WM: White matter | | | | | | |
